# Can we predict the future of respiratory failure prediction?

**DOI:** 10.1186/s13054-025-05484-7

**Published:** 2025-06-19

**Authors:** Alex K. Pearce, Shamim Nemati, Ewan C. Goligher, Catherine L. Hough, Andre L. Holder, Gabriel Wardi, Philip Yang, Aaron Boussina, Patrick G. Lyons, Sarina Sahetya, Atul Malhotra, Angela Rogers

**Affiliations:** 1 Division of Pulmonary, Critical Care, Sleep Medicine, and Physiology, University of California San Diego, La Jolla, CA USA; 2 Department of Biomedical informatics, University of California San Diego, La Jolla, CA USA; 3 Interdepartmental Division of Critical Care Medicine, University of Toronto, Toronto, ON Canada; 4 Department of Physiology, University of Toronto, Toronto, ON Canada; 5 Department of Medicine, Division of Respirology, University Health Network, Toronto, ON Canada; 6 Department of Medicine, Oregon Health & Science University, Portland, OR USA; 7 Division of Pulmonary, Allergy, Critical Care and Sleep Medicine, Emory University, Atlanta, GA USA; 8 Division of Critical Care, Department of Emergency Medicine, University of California San Diego, San Diego, CA USA; 9 Division of Pulmonary, Allergy, and Critical Care Medicine, Oregon Health & Science University, Portland, OR USA; 10 Division of Pulmonary and Critical Care Medicine, Johns Hopkins School of Medicine, Baltimore, MD USA; 11 Division of Pulmonary, Allergy, and Critical Care Medicine, Stanford University, Stanford, CA USA; 12 9500 Gilman Drive, 92093 La Jolla, CA USA

**Keywords:** Machine learning, Deep learning, Artificial intelligence, Acute respiratory failure, Invasive mechanical ventilation, Respiratory failure

## Abstract

**Background:**

Mortality in patients with acute respiratory failure remains high. Predicting progression of acute respiratory failure may be critical to improving patient outcomes. Machine learning, a subset of artificial intelligence is a rapidly expanding area, which is being integrated into several areas of clinical medicine. This manuscript will address the knowledge gap in predicting the onset and progression of respiratory failure, provide a review of existing prognostic strategies, and provide a clinical perspective on the implementation and future integration of machine learning into clinical care.

**Main body:**

Existing strategies for predicting respiratory failure, such as prediction scores and biomarkers, offer both strengths and limitations. While these tools provide some prognostic value, machine learning presents a promising, data-driven approach to prognostication in the intensive care unit. Machine learning has already shown success in various areas of clinical medicine, although relatively few algorithms target respiratory failure prediction specifically. As machine learning grows in the context of respiratory failure, outcomes such as the need for invasive mechanical ventilation and escalation of respiratory support (e.g. non-invasive ventilation) have been identified as key targets. However, the development and implementation of machine learning models in clinical care involves complex challenges. Future success will depend on rigorous model validation, clinician collaboration, thoughtful trial design, and the application of implementation science to ensure integration into clinical care.

**Conclusion:**

Machine learning holds promise for optimizing treatment strategies and potentially improving outcomes in respiratory failure. However, further research and development are necessary to fully realize its potential in clinical practice.

## Background/Introduction

Mortality among patients requiring invasive mechanical ventilation for acute respiratory failure remains high [1]. Several observations suggest that the ability to predict progression of respiratory failure may improve outcomes in these patients. Recent data underscore several important points. First, delayed intubation is associated with an increase in mortality [2]. Second, prolonged invasive mechanical ventilation is associated with higher costs, morbidity, and mortality [3]. Beyond financial costs, invasive mechanical ventilation imposes substantial psychosocial burdens and other complications stemming from prolonged critical illness [4]. On the other hand, identifying patients at very low risk of respiratory failure may also be useful as they may be better served outside of the Intensive Care Unit (ICU) or even outside of the hospital. Predictive analytics offer a promising solution to optimize resource utilization and potentially improve patient-centered outcomes in respiratory failure. However, current methods to predict respiratory failure onset and progression remain limited, with only moderate accuracy [5–7]. This gap highlights the need for further research in this area.

**Fig. 1:**
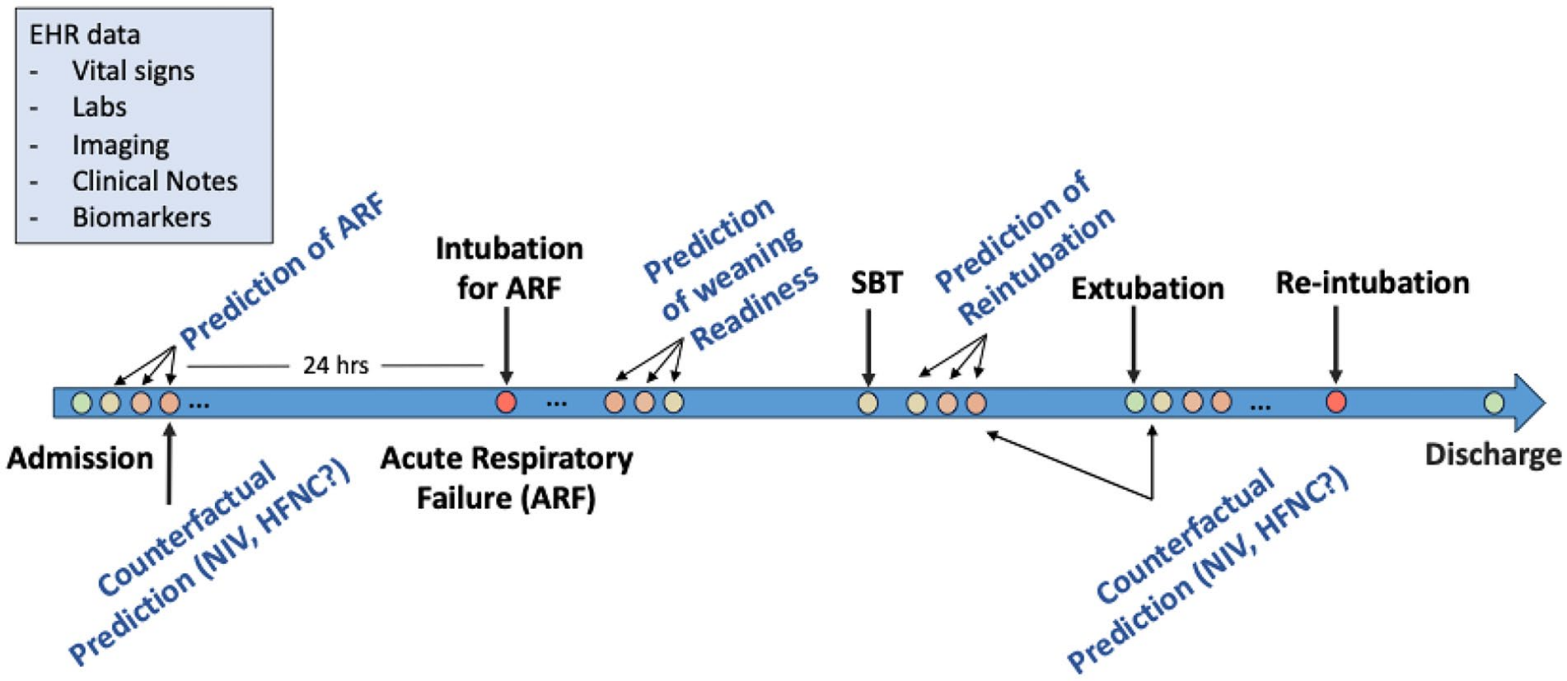
Timeline of possible impact of deep learning algorithms to influence clinical progression of patients with respiratory failure. Counterfactual models can be used to assess “what if” scenarios to prevent intubation or reintubation as a possible means to guide clinical management. Abbreviations: NIV = Noninvasive ventilation; HFNC = High flow nasal cannula; EHR = Electronic health record; SBT = Spontaneous breathing trial

Machine learning (ML), a rapidly advancing subset of artificial intelligence (AI), offers a considerable opportunity. ML enables the ingestion of vast amounts of data and has the potential to outperform existing approaches at predicting respiratory failure (Fig. 1). To explore this potential, we convened a panel of experts to review the current literature regarding predictors of respiratory failure and discussed the potential for ML applications to aid in the prediction of respiratory failure onset and progression. We chose the panel members based on contributions to the published literature (in artificial intelligence, mechanical ventilation, respiratory physiology, ICU trial design etc.) but also attempted to achieve geographic as well as demographic diversity among participants. Our goal was not an exhaustive systematic review but rather focus on key topics that our expert panel felt were most critical in this area. The panel’s discussion focused on several key areas: (1) A review of established strategies, such as prediction scores and biomarkers, to predict respiratory failure and related outcomes (Table 1). (2) The current state of ML in predictive analytics for medicine. (3) Future objectives for ML in respiratory failure prediction, including outcomes of interest, model development, implementation, and approaches to trial design. (4) Health equity considerations in ML applications.

**Table 1:** Examples of existing tools/models for predicting respiratory failure

Prognostic tool/model	Brief description	Limitations	Reference
Prognostic scores/indices			
Lung Injury Prediction Score (LIPS)	Prognostic score based on demographic and clinical data from ED patients at time of admission to predict development of Acute Lung Injury*	Works in limited clinical settings (at time of admission but not after; includes many variables leading to challenges in calculation	Trillo-Alvarez et al. Eur Respir J 2011 [5] Gajic et al. AJRCCM 2011 [8]
ROX Index	Simple index calculated from (SpO_2_/FiO_2_]/Respiratory Rate) to predict IMV in ICU patients on HFNC	Limited to ICU patient population already using HFNC	Roca et al. J. Crit Care 2016 [6]
Biomarkers			
Plasma biomarkers e.g. IL6, Ang 2, RAGE, lactate	Plasma samples prognostic in ARDS	Limited data in predicting respiratory failure before established; not always accessible clinically; need serial sampling studies	Agarawal et al. AJRCCM 2013 [22]
Arterial Blood Gas values: e.g. PaCO_2_/PaO_2_	Arterial blood gas readily available in ICU	Identify respiratory failure rather than predict it	Qadir et al. AJRCCM 2024 [75]
Artificial Intelligence/Machine Learning models			
VentNet	Deep learning algorithm using electronic health record showing robust Area Under Curve	Not clear how actionable for prevention; barriers to implementation; requires prospective validation multicenter	Shashikumar et al. Chest 2021 [46]

*The term Acute Lung Injury was phased out following the release of the Berlin ARDS criteria in 2012 and has been replaced with the term mild acute respiratory distress syndrome.

IMV = Invasive Mechanical Ventilation; HFNC = High Flow Nasal Cannula; IL-6 = interleukin 6; Ang 2 = angiotensin 2; RAGE = receptor for advanced glycation end products;

## Traditional predictors of respiratory failure

There are several existing prognostic scores/indices to predict outcomes related to respiratory disease including the development of Acute Respiratory Distress Syndrome (ARDS), failure of high flow nasal cannula (HFNC) support or respiratory failure progression despite non-invasive ventilation (NIV), and risk of need for invasive mechanical ventilation. The Lung Injury Prediction Score (LIPS), developed in 2011, was designed to identify patients at high risk for the development of acute lung injury during hospital admission using 25 variables including demographic, laboratory, and vital data [5, 8]. The score performed well at the time of patient admission from the ED to the hospital; however, in patients already admitted to the hospital, the Lung Injury Prediction score did not perform as well, limiting its applicability beyond the initial ED cohort [9].

The ROX index, another frequently cited scoring system, first described in 2016 [6] with subsequent multicenter validation in 2019 [10], is a simple tool used to predict the need for invasive mechanical ventilation in ICU patients with pneumonia on high-flow nasal cannula (HFNC). The ROX index is calculated as ([SpO_2_/FiO_2_]/Respiratory Rate) [6]. At 12 h following HFNC initiation, a ROX index > 4.88 correlates with a lower risk of intubation, while a value < 3.85 indicates a higher risk [10]. A subsequent meta-analysis showed an AUC of 0.81 for predicting intubation [11]. More recently, the VOX index was introduced, while similar to the ROX index, the VOX index replaced the respiratory rate variable with tidal volume [7]. The authors proposed that tidal volume is a more sensitive indicator of changes in respiratory drive than respiratory rate [7]. Tidal volume measurements were obtained during a brief NIV trial in patients treated with HFNC [7]. Evaluated in a small single-center study, the VOX index outperformed the ROX index and provided greater discriminatory power earlier after HFNC initiation at the 0, 2, and 6-hour windows [7]. Despite the ease of bedside calculation, these indices target a narrow patient population, only including patients with HFNC as their oxygen delivery modality. They also provide information on a patient who is already at high-risk, e.g. on high amounts of respiratory support (HFNC) and requiring ICU admission.

More complicated scoring systems also exist, examining a wider range of respiratory pathologies and oxygen delivery modalities. For example, the HACOR score predicts the risk of mechanical ventilation after the initiation of NIV in patients with acute hypoxemic respiratory failure of any etiology [12]. The HACOR score incorporates heart rate, acidosis (pH), consciousness (GCS), oxygenation (PaO_2_/FiO_2_ ratio), and respiratory rate. At 1-hour, a HACOR score > 5 is associated with a high risk of NIV failure with an AUC 0.71 [12]. Refinements to the HACOR score, adding six additional variables— sequential organ failure assessment (SOFA) score, presence of pneumonia, septic shock, cardiogenic pulmonary edema, ARDS, and immunosuppression—improved its predictive capabilities, raising the AUC to 0.78 [13]. Despite the earlier prognostication time (1–2 h after initiation of NIV) offered by the HACOR score compared to other prediction scores, its limitations include the scoring system complexity and number of variables required for calculation. Additionally, the use of NIV has decreased in the pneumonia/ARDS population following the High-Flow Oxygen through Nasal Cannula in Acute Hypoxemic Respiratory Failure (FLORALI) trial, which demonstrated a 90-day mortality benefit of HFNC over NIV in acute hypoxemic non-hypercapnic respiratory failure [14].

Expert panel members agreed that these scores are rarely used in clinical practice. Lack of routine use of existing scoring systems may be due to multiple factors. Some highlighted limitations include that current algorithms can identify, but not necessarily predict, respiratory failure. Instruments which rely on tachypnea and hypoxemia are likely identifying established or impending respiratory failure but not truly predicting a future event. Instead, expertise and clinical gestalt are heavily relied upon when assessing a patient and their risk of respiratory failure progression. However, it was recognized that even experienced clinicians provide incomplete evaluations based on variable time at the bedside, particularly overnight, with a high patient census, and may not be aware of decompensating patients outside the ICU setting in the absence of effective warning systems. There may also be some value in these indices in early medical education to help learners contextualize and form their own mental frameworks for understanding disease and illness severity.

### Role for biomarkers in respiratory failure predictions

PaO_2_ and PaCO_2_ are plasma biomarkers that are widely used to identify patients with impending respiratory failure and are embedded in many of the prognostic scores noted above. These biomarkers are also tested frequently and change over a short period of time, making them useful for identifying high-risk patients.

In contrast, other plasma biomarkers in respiratory failure have focused not on the prediction of respiratory failure but rather prognostic enrichment of patients with established respiratory failure, particularly ARDS [15, 16]. These plasma biomarkers focus on known pathways of ARDS and sepsis pathogenesis including inflammation [e.g. Tumor necrosis factor receptors (TNFR), Interleukin-6 (IL-6), Interleukin-8, Interleukin-18)], endothelial damage (e.g. Ang2), and epithelial damage [e.g. receptor for advanced glycation end products (RAGE) and Surfactant Protein D (SPD)].These ARDS biomarkers also define high-risk endotypes of disease (e.g. the “Hyperinflammatory class” of ARDS identified by Latent Class Analysis and other ML approaches in numerous cohorts and associated with differential response to therapies [17–20], or IL-18 which similarly identifies high-risk subset with a differential response to simvastatin) [21].

Despite a clear role for biomarkers in prognosis and endotyping of established ARDS, the role of these biomarkers *prior to the onset* of respiratory failure is less established. While the same inflammatory, epithelial, and endothelial biomarkers could serve a prognostic role in not-yet-critically ill patients, this has not been rigorously tested to date. Multiple ARDS biomarkers have been studied in large cohorts of critically ill patients with sepsis and/or ARDS and are higher in patients with ARDS than septic controls, suggesting potential prognostic value, but this is not the same as rigorous longitudinal sampling [17]. Few biomarkers have been tested in patients that initially presented without ARDS but later developed the condition, with Ang2 being an exception [22]. In the COVID-19 pandemic, large cohorts of patients with SARS-CoV-2 pneumonia who presented prior to progression to ARDS or respiratory failure were enrolled in biobanking studies or clinical trials. In these cohorts, RAGE and viral antigen levels have both been highly associated with progression to respiratory failure [23–25]. Most plasma biomarkers are measured at single time points or with several days between measurements; shorter interval trends have not been tested for rapidly developing respiratory failure, which is more classically seen in non-COVID pneumonias. Additionally, many of these biomarkers are primarily research based and not yet available for rapid testing in most clinical laboratories limiting their ability to guide clinical management in real-time.

While lung-specific samples like exhaled breath are already established biomarkers in other pulmonary diseases [e.g. exhaled NO levels as a marker for inflammation in asthma [26]] they are less studied in patients with impending respiratory failure. Elevated volatile organic compounds (VOCs) have been associated with pneumonia in multiple cohorts [27], but most of these studies focus on intubated patients. In non-intubated patients on high levels of respiratory support, there may be technical challenges in measuring VOCs, for example, a patient on HFNC may have difficulty performing testing to capture this sample adequately. Bronchoalveolar lavage (BAL) or HME filter testing can identify signatures of high-risk patients but can be challenging to obtain in non-intubated patients. Thus, the potential role of such lung specific samples in identifying high-risk patients is not established despite mechanistic promise. A synergistic approach between ML and biomarkers (e.g. ML model identifies a high-risk patient and triggers more frequent breath and plasma biomarker collection, which in turn informs ML risk score) may offer a powerful approach for risk prediction.

## Machine learning in critical care

Over the past 10 years, interest in the application of machine learning (ML) methods in clinical medicine has substantially increased [28, 29]. Machine learning techniques train computers to do what comes naturally to humans: learning by example. ML models use digitized data (input features) to make predictions for specified outcomes (or outputs/labels). Deep learning (DL), a branch of ML, achieves this task by using layers of non-linear processing (artificial neural networks), to produce a distributed and hierarchical representation of input features [30]. The hospital is an environment rich with patient data, requiring the integration of multiple data sources (e.g., vital signs, imaging, labs, and provider notes). Given the breadth and wealth of patient data available, ML can compute complex interactions or temporal relationships among multivariate risk factors that clinicians may overlook [31]. Large language model (LLM) integration may also add valuable information by incorporating unstructured patient data, like clinical notes. Integrating information from clinical notes and structured EHR data may improve predictive capabilities [32] as well as enhance clinical decision making [33].

In modern healthcare systems, ML/DL algorithms can be applied to clinical decision support (CDS) systems [34]. Some examples of CDS systems include prediction algorithms for sepsis [35], acute kidney injury [36], physiological deterioration [37], 30-day unplanned hospital readmissions [38], and emergency department triage [39]. Additionally, ML models have been applied in acute respiratory failure, particularly during the invasive mechanical ventilation phase, for example to identify patient-ventilator asynchrony [40, 41], to optimize ventilator settings [42], and predict response to lung recruitment [43] and prone positioning [44].

Several deep learning models exist that predict respiratory failure in the hospital setting [45–49]. Models were trained to predict various outcomes, including predicting HFNC failure [47], NIV failure [48], and need for invasive mechanical ventilation (IMV) [46]. However, limitations to these models exist. These models were retrospective and few have been prospectively validated [45]. In some models, the timeframe between prediction and event onset was relatively short or predictions were only created at 4-hour intervals, potentially limiting ability to implement meaningful interventions [45, 49]. Outside of a handful of academic institutions, these models are rarely integrated into clinical workflow. Barriers to implementation include challenges in analytic platform integration into the electronic health record (EHR) [50], costs to the health system [51], clinician acceptance [52], and model maintenance over time and among different patient populations and clinical practice variations [53]. Despite these challenges and limitations, these models provide an excellent starting point for ML models in the prediction of acute respiratory failure.

## Key objectives and outcomes for ML in respiratory failure

Part of the discussion focused on what respiratory outcomes are most critical. Panelists agreed that predicting emergence and progression of respiratory failure would be the most ‘actionable’. Specifically, panelists identified that the requirement for invasive mechanical ventilation was a critical outcome of interest. However, initiation of high flow nasal cannula (HFNC) and/or non-invasive ventilation (NIV) was also felt to be important, particularly if these therapies require transfer to the ICU. Local practice varies, as some centers require ICU transfer for initiation of therapy whereas others have intermediate care units which allow for these oxygen delivery modalities. The considerable variation across centers did not appear to be data driven but rather reflected local/regional practices and experiences, perhaps emphasizing the need for further study to inform such decisions. Additional potential outcomes of interest for future studies included both patient-related and health system-related [34]. Relevant patient related outcomes may include mortality, duration of mechanical ventilation, ICU length of stay (LOS), and tracheostomy rates among others [54]. For the health system, in addition to some parallel outcomes of interest (e.g. ICU LOS), system related outcomes such as cost, and resource utilization may also be of interest.

The panel also discussed several priorities for predictive analytics in the respiratory failure. Panelists emphasized the important impact of early knowledge of a patient’s likelihood of respiratory failure progression [e.g. nasal cannula (NC) to HFNC to IMV]. Providing advanced warning is most useful when it gives clinicians enough lead time to collect and interpret focused diagnostics and potentially intervene to prevent deterioration. There was debate regarding the optimal prediction horizon length (the time frame in which the outcome could occur or when prediction should apply), but most agreed that a 12 to 24-hours before the onset of respiratory failure would be a useful window to implement preventative strategies. This time frame would also allow the design of randomized controlled trials to test interventions to prevent progression of respiratory failure.

Another key element is clinical collaboration and incorporating appropriate risk thresholds that are important to clinical decision-making. For example, working with clinicians to identify appropriate risk thresholds for notification is crucial. This approach helps notify clinicians only at thresholds they believe to be clinically important, avoiding alarm fatigue and over-notification.

Predicting response to specific interventions, via methods such as counterfactual prediction [55], may also be of use and enhance the actionability of ML models and be applied to trial design. The approach of using counterfactual predictions allows a simulation of various different treatment responses. In other words, this approach answers the “what if” the patient were to receive HFNC scenario over another therapy. Such predictions can be used to inform clinical decision making and/or trial design and be locally adapted as needed. While predicting level of care may be valuable, there are regional practice variations in the use of respiratory modalities at different levels of care. It was generally agreed that predicting and tracking progression along the continuum of escalating respiratory support may be the most valuable and generalizable approach across systems. We recognize there may be variations in timing of endotracheal intubation across institutions; thus, strategies such as transfer learning and site-specific tuning will be essential. Transfer learning involves fine tuning of knowledge gained from a specific ML task or dataset is used to improve model performance on a similar task or different clinical site [56, 57].

## Challenges in ML model development, implementation and deployment

There are many challenges in the development, implementation, and deployment of risk related models in the clinical setting, especially for syndromic conditions like respiratory failure [58]. The development and validation of deep learning models can be challenging at both a patient level and a systems level. At the patient level, the diverse pathology underlying respiratory failure, different types of respiratory failure, and variability of treatment make model development challenging. Additionally, many of these patients experience concomitant organ failure, such as renal failure or cardiovascular failure, further complicating their clinical presentation. In contrast, existing models that have shown measurable clinical impact, such as the sepsis prediction model implemented by Boussina et al. [59] benefit from sepsis being a well-defined pathological construct with an established treatment paradigm.

Missing data and data heterogeneity, including inconsistencies in data sources, formats, frequency and quality, represent another major hurdle that can undermine the accuracy of ML models [60]. Specifically in respiratory failure, emergency procedures such as intubation and initiation of mechanical ventilation are not always well delineated or accurately time stamped in the EHR, leading to challenges with appropriate classification and model accuracy. At a systems level, there is substantial heterogeneity across health systems, regional practice patterns, and available resources. This variability complicates the creation of generalizable ML models [53]. For example, the decision to intubate may vary across institutions, thus a model developed at one institution may not perform well when tested at another with a different intubation threshold. Descriptions of different types of respiratory support may also vary, making it more difficult to accurately capture a patient’s condition from one hospital to another. Recent data suggest that ML approaches, such as transfer learning may mitigate this shortcoming [56]. Other groups are attempting to harmonize data capture to facilitate across system collaboration [61].

Beyond development challenges, there are several barriers to the successful implementation of these models into clinical care. Successful integration and deployment rely on several factors, including appropriate systems to ensure an efficient, adequate, and safe interface between the electronic health record and analytics platform, ensuring system performance, cost coverage, clinician buy-in, and a positive impact on patient-centered outcomes [62]. ICU physicians have expressed hesitancy surrounding the idea of deploying AI models in the ICU environment [52]. Avoiding “black box” models and promoting transparency in model determinants can help promote clinician acceptance and collaboration [63]. Fostering a symbiotic relationship between ML experts and clinicians is essential. Post-implementation, a robust system to monitor and iteratively improve model performance is necessary to avoid model drift [62].

## ML development and implementation into clinical practice and trial design

The successful integration of ML systems into clinical practice requires several steps, from creation to implementation, that involves diligent testing and validation. To advance ML integration in clinical settings, future research must focus on prospective randomized studies. However, several intermediary stages must be carefully addressed before reaching the point of prospective testing and validation. It is crucial to ensure that the models demonstrate adequate performance and maintain robust performance across systems and populations. One promising strategy is the use of “silent trials”, where the predictive model is integrated into the electronic health record but works in the background without interaction with end users [64]. Such strategies can allow enhancement of predictive abilities [64], identify bugs, and evaluate false positives and negatives, prior to full-scale clinical deployment.

Once a model is ready for prospective testing, several approaches can be considered for determining both what to test and how to evaluate its effectiveness. A key question is whether the risk score alone is sufficient or if it must be combined with an intervention or predicted treatment effect to impact patient outcomes meaningfully. The panel agreed that comparing model integration into clinical practice versus the existing standard of care is essential for evaluating its effectiveness.

There were several approaches suggested for future trial design. While single-center pilot trials may be an important approach early in ML validation due to their efficiency, thy may lack generalizability and require replication, as well as assessment of portability and scalability. Therefore, prospective, multicenter testing will likely be needed to garner widespread acceptance and ensure generalizability. While randomized controlled trials (RCTs) are often regarded as the gold standard, they are expensive and challenging to undertake. Alternative designs such as stepped-wedge or pragmatic trials may be more practical for testing the impact of incorporating ML models into clinical practice. Cluster randomized clinical trial designs may be more appropriate since these ML models are designed to intervene on the function of systems of care, not merely on mechanisms of injury in individual patients. The utility of predictive model could also be demonstrated by showing that treatment effects of interventions designed to prevent respiratory failure varies according to risk strata defined by predictive analytics. The panel acknowledged the role of different trial designs but emphasized that prospective, multicenter trials remain an important step towards broader acceptance.

The use of implementation science is also key. The implementation of new evidence-based practices within the healthcare setting is often challenging and can benefit from using standardized frameworks [65]. For example, approaches such as the Exploration, Preparation, Implementation, Sustainment (EPIS) framework can be used to facilitate implementation of ML in the clinical setting [65, 66]. Evaluation of implementation response and end-user interaction is crucial to understand better how clinicians interact with the algorithm and how to improve adoption and enhance user experience [67].

### Health equity

One concern with technological advancements in healthcare is patients who are socioeconomically advantaged may benefit disproportionately due to better access to resources and infrastructure that support successful implementation. Biased data or inconsistencies in the frequency of measurements may unintentionally promote disparities- systematic differences in model performance or predictive accuracy according to social characteristics [47, 68, 69]. However, ML algorithms, if designed correctly, could be used to achieve health equity rather than to worsen disparities. By relying on objective data accessible to all patients, models can be designed to for sex, race/ethnicity, religion, and skin color in an unbiased manner. A few noteworthy points must be considered in the context of a health equity discussion:

First, health systems with fewer resources, serving people with lower socioeconomic status, may be late to adopt new technologies which may further exacerbate existing health disparities and will need to be carefully considered.

Second, even when data are available, that data may be fundamentally biased. For example, skin color can influence the accuracy of some diagnostic methods, such as pulse oximetry [70, 71]. Accounting for differential accuracy of pulse oximetry based on skin color could help to improve its accuracy. Careful quantification of skin melanin using spectrophotometry can provide rigorous data, enabling adjustments to oximetry values if necessary. Technologies which are independent of skin color such as the measurement of tensions in exhaled gases (alveolar gas meter) could be used to avoid potential biases [72]. Alternatively, corrective factors may be applied for groups at-risk of bias related to pulse oximetry.

Third, natural language processing (NLP) or large language models can be quite helpful in analyzing progress notes by nurses, respiratory therapists, and physicians. However, implicit bias can influence subtle language patterns that are used preferentially for certain groups [73]. Refining NLP algorithms could help them recognize these biases and ensure that decisions are based on objective data rather than preconceived notions or historical tendencies.

Advocates for health equity emphasize the need to evaluate potential disparities in model performance and patient outcomes across different socioeconomic groups, promoting transparency in model development and deployment [74]. It will be important to address health disparities in the development of any ML model to ensure equitable performance and minimize bias.

## Conclusion

Prediction of respiratory failure progression is a critical topic that may benefit from the integration of ML models into clinical practice. ML offers powerful tools for predicting respiratory failure by integrating multimodal data to elucidate complex patterns that clinicians cannot always recognize. These models are particularly well-suited to the area of respiratory failure given the high number of data streams (compared to other areas of the hospital) and its resource intensive management. Additionally, current management of acute respiratory failure is often reactive. Enhancing predictive capabilities through ML could facilitate a more proactive approach to patient care, potentially improving outcomes. However, many challenges must be addressed to achieve meaningful integration into clinical care. These challenges include ensuring data consistency and quality, defining important outcomes, prospective implementation and validation of ML models, conducting clinical trials to evaluate their effectiveness, and ensuring equitable model performance across socioeconomic groups. Despite the challenges, ML offers a promising avenue to advance critical care and improve patient outcomes.

## Data Availability

No datasets were generated or analysed during the current study.

## Abbreviations

ML: machine learning
AI: artificial intelligence
ICU: Intensive Care Unit
HFNC: high flow nasal cannula
NIV: non-invasive ventilation
LIPS: lung injury prediction score
GCS: Glasgow coma score
SOFA: sequential organ failure assessment
ARDS: Acute Respiratory Distress Syndrome
DL: Deep Learning
CDS: clinical decision support
IMV: Invasive mechanical ventilation
NLP: natural language processing
